# Genomic Characterization of Selected Multidrug-Resistant *Escherichia coli* Isolates from Healthy Dogs in Chile Reveals Diverse Lineages Including ST131

**DOI:** 10.3390/ani16121769

**Published:** 2026-06-08

**Authors:** Fernando Sánchez, Nicolás Galarce, Leonardo Sáenz, Lisette Lapierre

**Affiliations:** 1 Departamento de Medicina Preventiva Animal, Facultad de Ciencias Veterinarias y Pecuarias, Universidad de Chile, Santiago 8820808, Chile; fernando.sanchez@uchile.cl (F.S.); ngalarce@uchile.cl (N.G.); 2 Programa de Doctorado en Ciencias Silvoagropecuarias y Veterinarias, Universidad de Chile, Santiago 8820808, Chile; 3 Departamento de Ciencias Biológicas, Facultad de Ciencias Veterinarias y Pecuarias, Universidad de Chile, Santiago 8820808, Chile; leosaenz@uchile.cl

**Keywords:** *Escherichia coli*, antimicrobial resistance, multidrug resistance, extended-spectrum β-lactamase, phylogenomics, ST131, One Health, companion animals, healthy dogs

## Abstract

Companion animals are increasingly recognized as carriers of antimicrobial-resistant *Escherichia coli*, yet genomic data from clinically healthy dogs in Latin America remain scarce. We characterized 13 genetically non-redundant multidrug-resistant *E. coli* isolates selected from 224 resistant *E. coli* isolates recovered in a previous surveillance study of 600 clinically healthy household dogs in the Metropolitan Region of Chile (2021–2022). Antimicrobial susceptibility testing was performed using VITEK2 and interpreted according to Clinical and Laboratory Standards Institute criteria, and whole-genome sequencing was used to identify resistance genes, virulence-associated traits, plasmid replicons, phylogroups, sequence types, and ST131 subclades. All isolates were multidrug resistant and showed high resistance to ampicillin and cephalexin (13/13, 100%), ciprofloxacin (13/13, 100%), cefpodoxime (11/13, 84.6%), cefovecin (10/13, 76.9%), and ceftiofur (10/13, 76.9%). An extended-spectrum β-lactamase phenotype was observed in 9/13 isolates (69.2%). *bla*_CTX-M_ genes were detected in 7/13 isolates (53.8%) and *bla*_CMY-2_ in 3/13 isolates (23.1%), whereas IncF-associated plasmid replicons predominated. The isolates belonged to diverse lineages, including ST131, ST744, ST1196, and ST1011. The two ST131 isolates belonged to phylogroup B2, carried *bla*_CTX-M_ variants, and were assigned to the C2/H30Rx and C1-M27 subclades. These findings support further consideration of companion animals in genomic antimicrobial resistance surveillance under a One Health framework.

## Simple Summary

Healthy dogs can carry intestinal *Escherichia coli* resistant to several antibiotics, but genomic information from Latin America remains limited. In this study, we analyzed 13 selected resistant *E. coli* isolates obtained from fecal samples of clinically healthy household dogs in Santiago, Chile. We combined antimicrobial susceptibility testing with whole-genome sequencing to describe the resistance genes, virulence-associated traits, plasmids, and genetic lineages present in these isolates. All selected isolates were resistant to multiple classes of antibiotics, and many were resistant to cephalosporins and fluoroquinolones that are important in veterinary and human medicine. Nine isolates showed an extended-spectrum beta-lactamase phenotype, and two belonged to sequence type 131, a globally distributed lineage well known in human medicine. These results suggest that clinically healthy dogs may carry resistant *E. coli* lineages of potential veterinary and public health relevance, supporting further consideration of companion animals in surveillance programs that track antibiotic resistance across people, animals, and the environment.

## 1. Introduction

Antimicrobial resistance (AMR) is widely recognized as one of the most pressing global health challenges, compromising the effective treatment of bacterial infections across human and veterinary medicine. Global estimates indicate that bacterial AMR was directly responsible for approximately 1.27 million deaths and associated with nearly 4.95 million deaths worldwide in 2019 [1], with modelling studies predicting a further increase in AMR-attributable mortality by 2050 if current trends persist [2]. The complex and interconnected nature of AMR has positioned it as a quintessential One Health problem, driven by the circulation of resistant bacteria and resistance determinants across human, animal and environmental interfaces [3,4,5].

Within this global context, resistant *Enterobacterales*, particularly *Escherichia coli*, have become central targets of international antimicrobial resistance surveillance and control efforts. The World Health Organization (WHO) recognizes third-generation cephalosporin-resistant and carbapenem-resistant *Enterobacterales* among the bacterial groups of highest public health concern. In parallel, European surveillance frameworks coordinated by the European Food Safety Authority (EFSA) and the European Centre for Disease Prevention and Control (ECDC) routinely monitor resistance in commensal and extended-spectrum β-lactamase (ESBL)/AmpC-producing *E. coli* from humans, animals and food [6,7]. Although international AMR mitigation efforts have historically focused on human healthcare and food-producing animals, accumulating evidence indicates that companion animals remain an underexplored component of AMR ecology, despite their close contact with humans and frequent exposure to critically important antimicrobials [5,8].

Because of its ubiquity and capacity to acquire resistance determinants, *E. coli* is widely regarded as a key indicator organism for AMR surveillance and has been explicitly incorporated into integrated surveillance frameworks, including the WHO Tricycle approach based on ESBL-producing *E. coli* across human, animal and environmental sectors [6,9]. In companion animals, particularly dogs, commensal *E. coli* constitutes a major component of the intestinal microbiota and has been repeatedly identified as a reservoir of multidrug-resistant (MDR) strains, including isolates resistant to critically important antimicrobials such as third-generation cephalosporins and fluoroquinolones [10,11,12,13].

Beyond resistance phenotypes, genomic characterization has become essential to assess the epidemiological relevance of *E. coli* circulating in companion animals. Extraintestinal pathogenic *E. coli* (ExPEC) represents a major public health concern due to its association with urinary tract infections, bloodstream infections and other invasive diseases in humans [14,15,16]. Although ExPEC is often discussed as a distinct pathotype, the boundary between ExPEC and commensal *E. coli* is not always clear, since many virulence-associated traits linked to extraintestinal pathogenicity can also occur in intestinal strains, and different combinations of genes may contribute to similar clinical outcomes [15,17]. Among ExPEC-associated lineages, sequence type (ST) 131 has emerged as a globally disseminated pandemic clone. This lineage is frequently linked to multidrug resistance, ESBL production, and the carriage of resistance and virulence determinants on epidemic IncF plasmids [18,19,20]. Importantly, ST131 and other high-risk ExPEC-associated lineages, including ST410, ST744 and ST1193, have also been reported in dogs and other companion animals across different geographic settings. These findings challenge the notion of strict host specificity and raise concerns regarding their broader ecological distribution [21,22,23].

Whole-genome sequencing (WGS) approaches, including core-genome phylogenetic and single-nucleotide polymorphism (SNP)-based analyses, have revealed close genetic relatedness between *E. coli* isolates recovered from humans and dogs, including lineages associated with extraintestinal pathogenicity, with shared STs, resistomes and plasmid architecture across host species [22,24,25,26]. These findings suggest that companion animals may participate in the circulation of high-risk *E. coli* lineages rather than harboring strictly host-adapted populations, reinforcing the need to integrate dogs into AMR surveillance frameworks under a One Health perspective [8,27].

Despite growing international evidence, genomic data on high-risk *E. coli* lineages and virulence-associated traits circulating in healthy dogs remain scarce in Latin America. In Chile, previous studies have documented antimicrobial-resistant *E. coli* in dogs from different epidemiological settings. In small-scale farms of central Chile, ESBL-producing *E. coli* carrying *bla*_CTX-M_ genes were detected among livestock, dogs and wild mammals, with dogs showing a higher fecal carriage frequency than livestock and wildlife [28]. In urban household dogs from the Metropolitan Region, previous studies based on the same surveillance framework documented phenotypic and genotypic antimicrobial resistance in *E. coli*, including the parent collection from which the isolates analyzed here were selected, and identified epidemiological risk factors associated with carriage of critical antimicrobial-resistant *E. coli* [13,29]. However, these previous analyses did not include WGS-based characterization of selected resistant canine *E. coli* isolates to define clonal lineages, plasmid replicons, virulence-associated repertoires and phylogenomic relationships. This limitation hampers the assessment of the epidemiological relevance of MDR *E. coli* from healthy dogs, particularly isolates belonging to lineages associated with extraintestinal pathogenicity, and constrains their integration into national and regional One Health surveillance frameworks. Therefore, the present study aimed to genomically characterize selected *E. coli* isolates recovered from healthy dogs in the Metropolitan Region of Chile. We focused on resistance and virulence-associated traits, plasmid backgrounds, and phylogenomic relationships, with particular emphasis on ST131 and other ExPEC-associated lineages of epidemiological interest. Building on a previously described phenotypic and epidemiological surveillance study of resistant *E. coli* from healthy dogs in Chile, this work focused on the whole-genome characterization of a targeted subset of genetically non-redundant MDR isolates selected to represent diverse genomic backgrounds.

## 2. Materials and Methods

### 2.1. Bacterial Strains and Selection Criteria

This study included 13 *E. coli* isolates recovered from fecal samples of clinically healthy household dogs sampled between 2021 and 2022 in veterinary clinics distributed across seven geographic sectors of the Metropolitan Region of Chile. These isolates originated from a previously described surveillance study involving 600 healthy dogs, from which 224 resistant *E. coli* isolates were recovered on antibiotic-supplemented MacConkey plates, as previously described by Galarce et al. [29].

For the present study, isolates were selected using a diversity-oriented rather than prevalence-based approach. The aim was to reduce clonal redundancy within the resistant *E. coli* collection and prioritize isolates with divergent molecular and resistance-related backgrounds for WGS. ERIC-PCR fingerprints generated during the parent surveillance study were used as a preliminary screening tool to identify non-identical banding profiles. Isolates were then prioritized considering divergent ERIC-PCR patterns together with available resistance information, including recovery from different antibiotic-supplemented media, resistance profiles, and selected resistance determinants. When isolates showed highly similar ERIC-PCR fingerprints and comparable resistance information, only one representative isolate was retained for sequencing. Thus, this panel should be interpreted as a genetically non-redundant subset intended to explore part of the phenotypic and genetic heterogeneity of the 224 resistant *E. coli* isolates, rather than as a proportional or prevalence-representative sample. Because ERIC-PCR was used as a preliminary fingerprinting method rather than a genome-scale population-structure approach, the proportion of total genomic diversity captured from the parent collection could not be formally quantified before WGS.

Dog-level metadata were retrieved from the original surveillance records and owner questionnaires when available. All isolates included in this study were obtained from clinically healthy household dogs sampled during routine veterinary visits or community recruitment, and all dogs fulfilled the parent study eligibility criterion of no recent antimicrobial treatment before sampling. Available metadata included dog/sample code, recruitment setting, geographic sector, commune, questionnaire availability, sex, age category, size, breed type, housing conditions, diet, and selected exposure variables. Because completed owner questionnaires were not available for all selected dogs, missing questionnaire-derived information is indicated as not available. Available dog-level metadata are provided in Supplementary Table S1.

### 2.2. Phenotypic Antimicrobial Susceptibility Testing

Phenotypic antimicrobial susceptibility of the 13 selected isolates was further characterized by determining the minimum inhibitory concentration (MIC) using the VITEK2 automated system (bioMérieux, Marcy-l’Étoile, France) with AST-GN98 cards, according to the manufacturer’s instructions. The panel included aminoglycosides, β-lactams, folate pathway inhibitors, nitrofurans, phenicols, quinolones, and tetracyclines. Isolates were classified as susceptible, intermediate, or resistant according to the CLSI VET01S 7th edition interpretive criteria published in 2024 [30]. For the purposes of this study, MDR was defined as resistance to three or more antimicrobial classes [31], and the multiple antimicrobial resistance (MAR) index was calculated as “a/b”, where “a” represents the number of antimicrobials to which the isolate was resistant and “b” the total number tested.

### 2.3. Whole-Genome Sequencing and Bioinformatic Analyses

Genomic DNA was extracted from the selected isolates using the Wizard^®^ Genomic DNA Purification Kit (Promega, Madison, WI, USA) according to the manufacturer’s instructions. DNA concentration and purity were assessed using a Nano-400 Micro-spectrophotometer (Hangzhou Allsheng Instruments Co., Ltd., Hangzhou, China). The extracted DNA was used for library preparation with the QIAseq FX DNA Library Kit (QIAGEN, Hilden, Germany) and paired-end sequencing was performed on an Illumina^®^ NextSeq 1000 platform (Illumina, San Diego, CA, USA), targeting a minimum genome coverage of 20×. This threshold was used as a minimum inclusion criterion for draft-genome analyses, whereas downstream analyses were performed only on assemblies that exceeded this threshold and met predefined quality metrics. The genomic analyses based on these assemblies were focused on gene detection, MLST, plasmid replicon screening and comparative phylogenomics, rather than complete plasmid reconstruction. Raw sequencing data were quality-checked using FastQC v0.12.0 and trimmed with Trimmomatic v0.39 [32] to remove adapter sequences and low-quality bases.

De novo genome assembly was performed using SPAdes v3.15.5 [33] in careful mode. Assembly quality was assessed using standard assembly statistics to ensure suitability for downstream comparative genomic analyses. Assemblies were filtered using quality thresholds of ≥200 bp minimum contig length and N50 >50 kb. Genome annotation was performed using Prokka v1.14.6 [34] and the NCBI Prokaryotic Genome Annotation Pipeline (PGAP) [35]. Antimicrobial resistance genes, virulence-associated genes, and plasmid replicons were identified using the Center for Genomic Epidemiology tools ResFinder v4.7.2 [36], VirulenceFinder v2.0 [37], and PlasmidFinder v2.1 [38], respectively, applying thresholds of ≥90% identity and ≥90% coverage. These conservative thresholds were selected to retain high-confidence gene and replicon calls, prioritize specificity in the final presence/absence matrices, and reduce potential false-positive calls from fragmented or low-similarity hits. However, this approach may reduce sensitivity and generate false-negative calls for highly divergent, truncated, or fragmented homologues. Therefore, the reported profiles should be interpreted as curated high-confidence detections rather than exhaustive screening of all possible resistance, virulence, or plasmid-associated homologues. These analyses were complemented with CARD (RGI v6.0.5, CARD v4.0.1) [39] and AMRFinderPlus v4.2.7 [40]. For manuscript reporting and figure construction, only curated presence/absence calls meeting the predefined thresholds were retained in the final binary matrix. Phylogroups, serotypes, fimbrial types, and STs were assigned using ClermonTyping [41], SerotypeFinder v2.0.1 [42], FimTyper [43], and the Achtman MLST scheme implemented in EnteroBase v1.2.0 [44]. Assembly statistics and the complete genomic features of the 13 isolates, including AMR genes, quinolone resistance-determining region (QRDR) mutations, plasmid replicons, and virulence-associated genes, are summarized in Supplementary Table S1.

### 2.4. Phylogenetic Analysis

Core genome single-nucleotide polymorphism (cgSNP)-based phylogeny was inferred using the CSI Phylogeny 1.4 pipeline [45] with *E. coli* K-12 MG1655 (RefSeq NC_000913.3) as the reference genome and default parameters, unless otherwise specified. Resulting phylogenetic trees were visualized and annotated using Interactive Tree of Life (iTOL v7) [46], incorporating metadata layers for sequence types, antimicrobial resistance genes, plasmid replicons, and sample origin.

Given the epidemiological relevance of ST131, isolates assigned to this lineage were subjected to expanded comparative phylogenomic analysis. Publicly available ST131 genomes were retrieved from EnteroBase [44], accessed in March 2026. At the time of retrieval, EnteroBase contained 29,992 *E. coli* ST131 genomes, of which 570 corresponded to South American entries. From this subset, genomes were retained when assembly FASTA files and associated epidemiological metadata, including BioProject/BioSample information, country of origin, and source, were available and when assembly quality metrics were comparable to those of the genomes generated in this study, including coverage >20× and draft assemblies suitable for comparative analysis. Genome assemblies lacking epidemiological metadata or not meeting predefined quality criteria were excluded from downstream analyses.

The final dataset comprised 170 public genomes after metadata and quality filtering. ST131 isolates identified in this study were analyzed together with the selected public genomes and the reference genomes EC958 and NCTC13441 for phylogenetic inference. Comparative core genome phylogenetic reconstruction of *E. coli* ST131 was then performed using a k-mer-based approach implemented in Split K-mer Analysis (SKA v0.5.1) [47]. Genome assemblies were converted into split-k-mer sketches and merged to generate a core genome alignment. Pairwise SNP distances were calculated using snp-dists v0.8.2. Maximum-likelihood phylogenetic inference was conducted using IQ-TREE v2.2.0 [48] with automatic model selection and ultrafast bootstrap approximation (1000 replicates). Resulting phylogenetic trees were visualized and annotated using iTOL v7. To further characterize the population structure of ST131 isolates, in silico subclade typing was performed using ST131Typer v1.0 [49], which assigns isolates to established ST131 sublineages based on diagnostic targets and *fimH* allele profiles.

## 3. Results

### 3.1. Isolate Characteristics and Dog-Level Metadata

The 13 selected *E. coli* isolates were recovered between 2021 and 2022 from clinically healthy household dogs attending veterinary clinics located in different sectors of the Metropolitan Region of Chile (Table 1). This isolate set corresponded to 12 unique dog/sample records because two isolates (AMX 16A and CEF 16B) shared the same dog/sample code. All dogs were clinically healthy household dogs and fulfilled the parent study eligibility criterion of no recent antimicrobial treatment before sampling. Completed owner questionnaires were available for 6/12 dog/sample records. Among records with available questionnaire data, three corresponded to females and three to males; four dogs were 2–10 years old and two were younger than 2 years; three were small, two were medium-sized and one was large. Three dogs were mixed-breed and three were purebred, including one Poodle Toy, one Shiba Inu and one German Shepherd. Additional non-identifiable dog-level metadata, including recruitment setting, commune, housing conditions, diet and selected exposure variables, are provided in Supplementary Table S1. In silico MLST analysis revealed a heterogeneous population structure that included ST131, together with ST10, ST90, ST162, ST224, ST491, ST744, ST1011 and ST1196, distributed across several phylogroups. Serotype and *fimH* assignments further underscored the genomic diversity represented within this panel. Consistent with the diversity-oriented selection strategy, the selected panel included nine STs and six phylogroups, supporting broad heterogeneity among the sequenced isolates, although it was not designed to quantify lineage frequencies in the parent collection. All 13 genomes passed the predefined assembly quality criteria for downstream analyses. Sequencing depth ranged from 28.5× to 112.3×, with a mean depth of 75.7×. Assembly sizes ranged from 4.80 to 5.36 Mb, N50 values ranged from 88,178 to 255,974 bp, and the number of contigs ≥200 bp ranged from 65 to 313. Detailed assembly statistics are provided in Supplementary Table S1.

### 3.2. Antimicrobial Resistance Phenotypes

**Figure 1:**
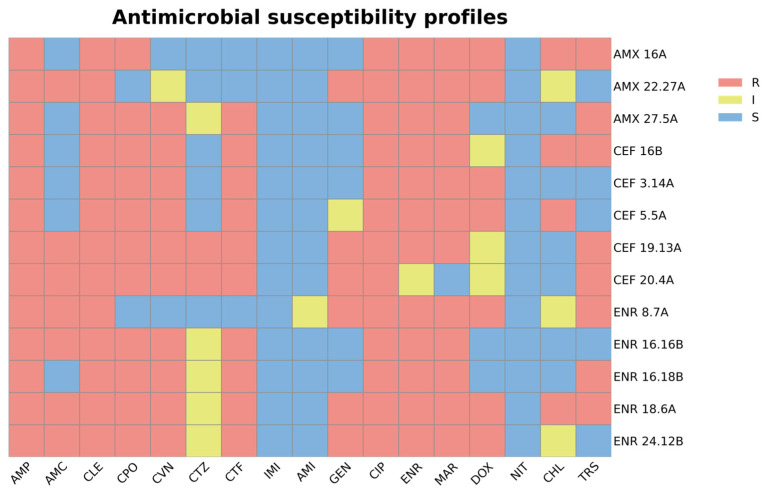
Heatmap of antimicrobial susceptibility profiles of 13 *E. coli* isolates recovered from healthy dogs in the Metropolitan Region of Chile. Colors represent susceptibility categories (blue = susceptible, yellow = intermediate, red = resistant) based on CLSI breakpoints. Rows correspond to isolates, shown in the same order as in Table 1, and columns correspond to antimicrobial agents organized by pharmacological class to facilitate comparison across isolates. The heatmap highlights a shared MDR pattern characterized by resistance to ampicillin, cephalexin and ciprofloxacin in all isolates, frequent resistance to third-generation cephalosporins and fluoroquinolones, and conserved susceptibility to imipenem and nitrofurantoin. Antimicrobial abbreviations and classes: AMP, ampicillin; AMC, amoxicillin/clavulanic acid (penicillins ± β-lactamase inhibitor); CLE, cephalexin (first-generation cephalosporin); CPO, cefpodoxime; CVN, cefovecin; CTZ, ceftazidime; CTF, ceftiofur (third-generation cephalosporins); IMI, imipenem (carbapenem); AMI, amikacin; GEN, gentamicin (aminoglycosides); CIP, ciprofloxacin; ENR, enrofloxacin; MAR, marbofloxacin (fluoroquinolones); DOX, doxycycline (tetracycline); NIT, nitrofurantoin (nitrofuran); CHL, chloramphenicol (phenicol); TRS, trimethoprim/sulfamethoxazole (folate-pathway inhibitor).

All 13 selected isolates displayed MDR phenotypes (Figure 1). Resistance to β-lactams was widespread, with all isolates resistant to ampicillin (13/13, 100%) and cephalexin (13/13, 100%). Resistance to third-generation cephalosporins was also frequent, including cefpodoxime (11/13, 84.6%), cefovecin (10/13, 76.9%) and ceftiofur (10/13, 76.9%). Fluoroquinolone resistance was prominent, with all isolates resistant to ciprofloxacin (13/13, 100%) and high resistance rates to enrofloxacin (12/13, 92.3%) and marbofloxacin (12/13, 92.3%). In contrast, susceptibility was largely retained for amikacin (12/13, 92.3%), whereas all isolates were susceptible to nitrofurantoin (13/13, 100%) and imipenem (13/13, 100%). An ESBL phenotype was detected in 9/13 isolates (69.2%). The MAR index ranged from 0.47 to 0.76, with a mean value of 0.58, further reflecting broad resistance across multiple antimicrobial classes among the selected isolates.

### 3.3. Resistome and Plasmid Replicons

**Figure 2:**
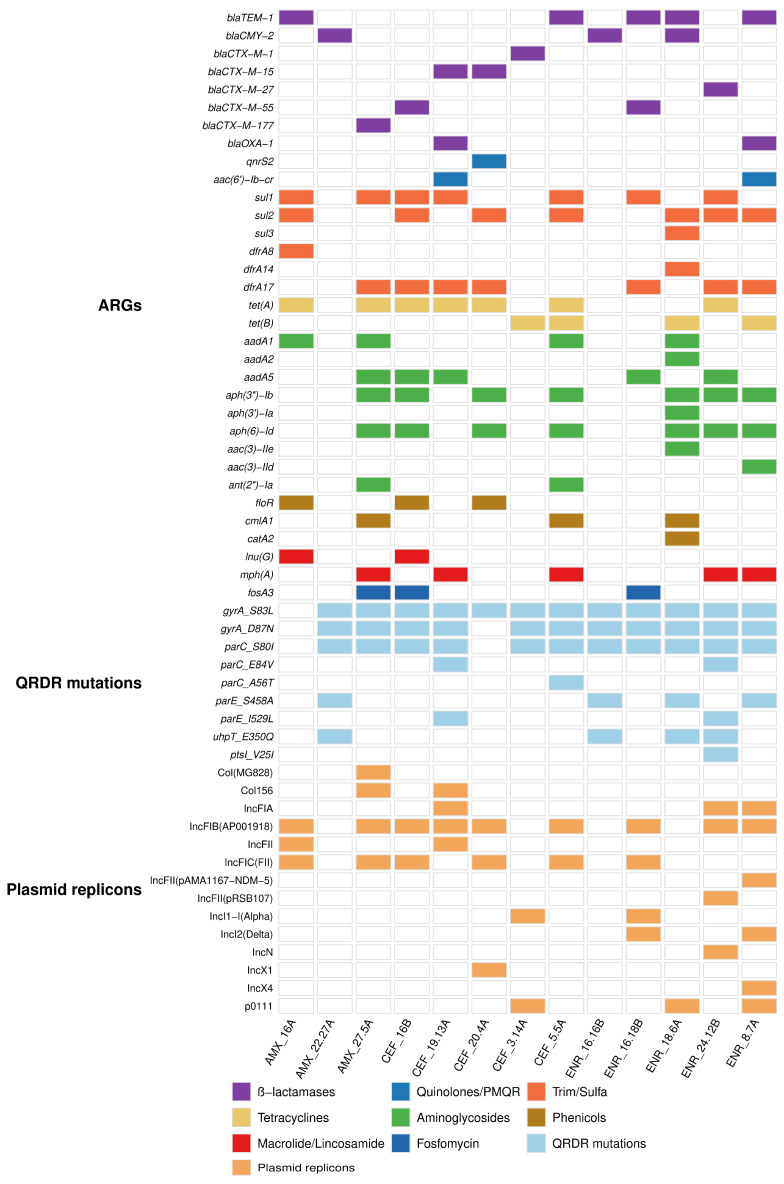
Matrix representation of curated antimicrobial resistance determinants, chromosomal resistance-associated mutations and plasmid replicons identified in 13 *E. coli* isolates recovered from healthy dogs in the Metropolitan Region of Chile. Rows represent resistance genes, resistance-associated mutations and plasmid replicon types, and columns correspond to individual isolates. The presence or absence of each feature is indicated for each isolate. Presence/absence calls shown in the matrix correspond to curated hits retained using thresholds of ≥90% identity and ≥90% coverage. Colors assigned to antimicrobial resistance genes reflect the antimicrobial class to which resistance is conferred. Key patterns highlighted by the matrix include the frequent occurrence of *bla*_CTX-M_ variants, the detection of *bla*_CMY-2_ in non-B2 lineages, the widespread presence of QRDR mutations associated with fluoroquinolone resistance, and the predominance of IncF-associated plasmid replicons. Together, these findings indicate the convergence of β-lactam and fluoroquinolone resistance determinants with plasmid backgrounds commonly associated with MDR dissemination. ARGs, antimicrobial resistance genes; QRDR, quinolone resistance-determining region; PMQR, plasmid-mediated quinolone resistance.

WGS revealed a diverse resistome underlying the observed phenotypes (Figure 2). ESBL genes of the *bla*_CTX-M_ family were detected in 7/13 isolates (53.8%), including *bla*_CTX-M-1_, *bla*_CTX-M-15_, *bla*_CTX-M-27_, *bla*_CTX-M-55_ and *bla*_CTX-M-177_. Both ST131 isolates carried *bla*_CTX-M_ variants (*bla*_CTX-M-15_ and *bla*_CTX-M-27_), whereas *bla*_CTX-M-55_ and *bla*_CTX-M-177_ were detected in non-B2 lineages, including ST1011 and ST162. In addition, the plasmid-mediated *bla*_CMY-2_ gene was identified in 3/13 isolates (23.1%), all belonging to non-B2 phylogroups.

Beyond β-lactam resistance, plasmid-mediated quinolone resistance genes were detected in 3/13 isolates (23.1%), including *qnrS2* (1/13) and *aac(6′)-Ib-cr* (2/13). Chromosomal QRDR mutations were also common, particularly substitutions in *gyrA* (S83L and D87N) and *parC* (S80I, and in some isolates E84V), whereas additional *parE* substitutions were less frequent (Figure 2). The combined presence of these QRDR alterations was consistent with the widespread ciprofloxacin resistance observed in this isolate set, beyond the contribution of plasmid-mediated quinolone resistance alone. Tetracycline resistance genes were common, with *tetA* detected in 7/13 isolates (53.8%) and *tetB* in 4/13 isolates (30.8%). Resistance to sulfonamides and trimethoprim was also frequent, with *sul1* and *sul2* each present in 7/13 isolates (53.8%) and *dfrA17* detected in 7/13 isolates (53.8%). The macrolide resistance gene *mph(A)* was identified in 5/13 isolates (38.5%), often co-occurring with ESBL and other resistance determinants.

Plasmid replicon analysis revealed a heterogeneous plasmidome, with a predominance of IncF-associated replicons (Figure 2). The most frequent replicons were IncFIB(AP001918), detected in 9/13 isolates (69.2%), and IncFIC(FII), detected in 6/13 isolates (46.2%), followed by IncFIA in 3/13 isolates (23.1%). IncFII and related IncFII variants were detected at lower frequencies. Additional non-IncF replicons, including Col-type replicons, IncI1-I(Alpha), IncI2(Delta), IncN, IncX1, IncX4 and pO111, were detected sporadically.

Overall, the integrated resistome and plasmidome profiles were broadly consistent with the phenotypic resistance patterns shown in Figure 1, with several isolates concentrating multiple resistance determinants and plasmid replicons, whereas others displayed narrower, but still clinically relevant, MDR profiles. A complete list of AMR genes, QRDR mutations and plasmid replicons identified in each isolate is provided in Supplementary Table S1.

To further relate genotype and phenotype, phenotypic resistance by antimicrobial class was compared with the corresponding antimicrobial resistance determinants detected by WGS (Supplementary Table S4). Resistance to third-generation cephalosporins was generally supported by the presence of *bla*_CTX-M_ variants and/or *bla*_CMY-2_, whereas the widespread ciprofloxacin resistance was consistent with QRDR mutations in *gyrA* and *parC*, with additional PMQR determinants detected in a subset of isolates. Tetracycline and folate-pathway resistance were supported by the detection of *tetA/tetB* and *sul1/sul2/dfrA17* determinants, respectively, where present. Overall, the genotype–phenotype comparison showed broad concordance, although isolated discrepancies may reflect gene expression, additional chromosomal mechanisms, regulatory changes, porin alterations, or limitations associated with short-read assembly and database-based detection.

### 3.4. Virulence-Associated Genes and ExPEC-Related Traits

**Figure 3:**
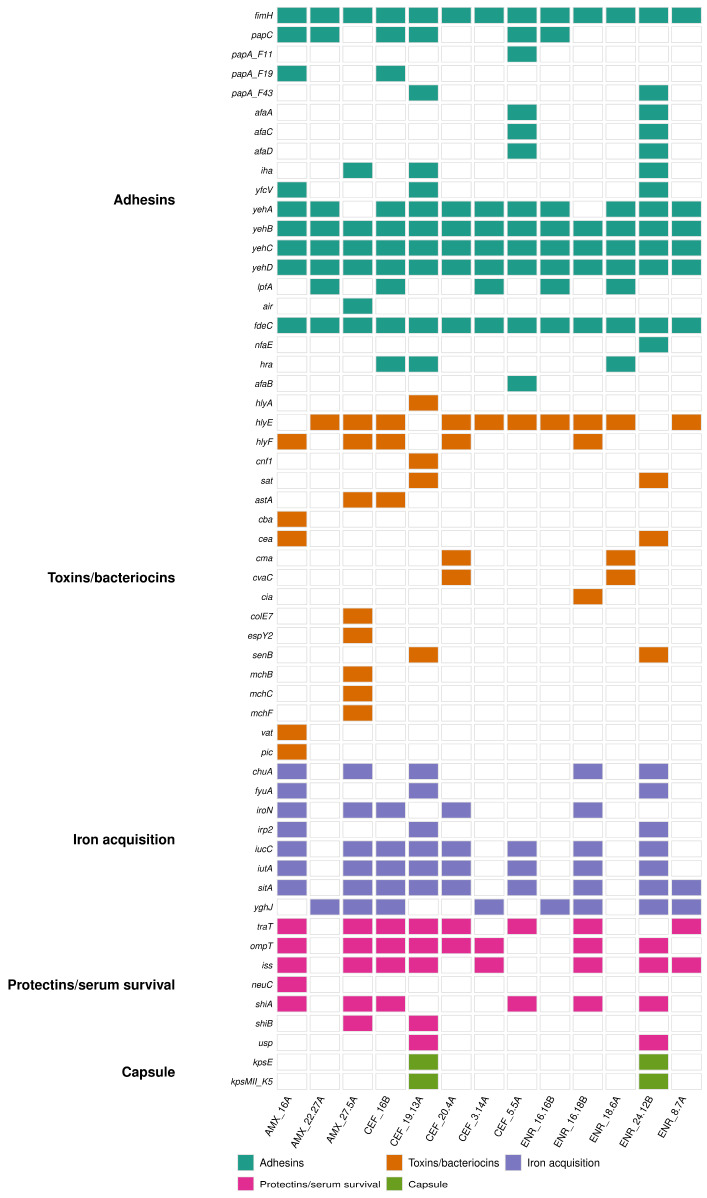
Matrix representation of curated virulence-associated genes detected in 13 *E. coli* isolates recovered from healthy dogs in the Metropolitan Region of Chile. Rows represent virulence-associated genes, and columns correspond to individual isolates. The presence or absence of each gene is shown per isolate. Presence/absence calls shown in the matrix correspond to curated hits retained using thresholds of ≥90% identity and ≥90% coverage. Colors indicate functional categories of virulence factors, including adhesion, iron acquisition, toxins/bacteriocins, serum resistance/protective factors and capsule-associated loci. The matrix highlights the heterogeneous distribution of virulence-associated genes, with broader ExPEC-associated repertoires concentrated mainly in B2/ST131 isolates, whereas most non-B2 lineages showed more variable and generally narrower accessory virulence profiles. This pattern suggests that selected fecal isolates carried virulence-associated traits of potential epidemiological relevance, particularly within B2/ST131 backgrounds.

Virulence gene profiling revealed marked heterogeneity among the 13 *E. coli* isolates (Figure 3). Adhesion-associated genes were broadly distributed, with *fimH* detected in all isolates (13/13, 100%), consistent with its role as a common colonization factor in pathogenic *E. coli*. Several additional adhesin-associated loci, including *pap*-, *afa*-, *yeh*-, *yfcV*- and *fdeC*-related genes, showed variable distribution across the isolate set.

Iron acquisition and serum survival systems frequently associated with extraintestinal pathogenicity were unevenly distributed. Genes related to the aerobactin system (*iucC* and *iutA*) were detected in 8/13 isolates (61.5%), whereas *iroN* was present in 5/13 isolates (38.5%). Other iron acquisition-associated loci, including *sitA*, *chuA*, *fyuA* and *irp2*, also showed variable distribution across the isolate set. Serum survival-associated genes were common in a subset of isolates, particularly *traT*, *ompT* and *iss*, each detected in 8/13 isolates (61.5%). Capsule-associated loci (*kpsE* and *kpsMII_K5*) were restricted to 2/13 isolates (15.4%), both belonging to ST131 backgrounds.

Toxin- and bacteriocin-associated genes were also heterogeneously distributed. *hlyE* was the most frequent toxin-associated gene, being detected in 10/13 isolates (76.9%), whereas *hlyF* was present in 5/13 isolates (38.5%). In contrast, *hlyA* and *cnf1* were each detected in only 1/13 isolates (7.7%), both in the ST131 isolate CEF 19.13A. Other toxin- and bacteriocin-associated loci, including *sat*, *astA*, *cma*, *cvaC*, *cea* and *senB*, were detected sporadically.

Overall, virulence-associated traits linked to extraintestinal pathogenicity were concentrated in a subset of isolates, particularly within B2 backgrounds, whereas most non-B2 lineages displayed narrower but still epidemiologically relevant accessory virulence profiles. The complete virulence-associated gene profiles of the 13 isolates are summarized in Supplementary Table S1.

### 3.5. Phylogeny and High-Risk Lineages

**Figure 4:**
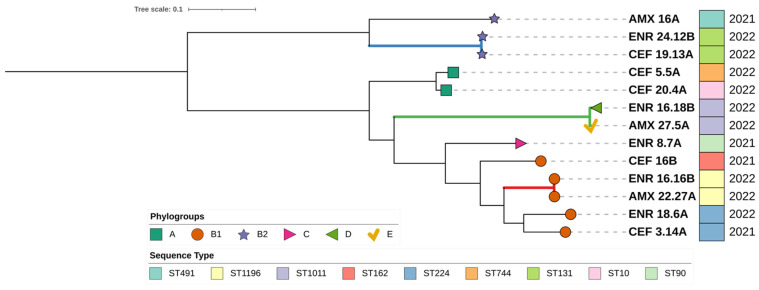
Core-genome single-nucleotide polymorphism (cgSNP)-based phylogeny of 13 multidrug-resistant (MDR) *E. coli* isolates recovered from healthy dogs in the Metropolitan Region of Chile. The phylogenetic tree was inferred using the CSI Phylogeny pipeline with *E. coli* K-12 MG1655 as the reference genome. The tree is based on a core-genome SNP alignment, and branch lengths represent nucleotide substitutions per site. Symbols and colors indicate phylogroup, sequence type and year of isolation, as shown.

The cgSNP phylogenetic analysis of the 13 canine isolates revealed a structured population consistent with MLST and phylogroup assignments (Figure 4). The two ST131 isolates, both belonging to phylogroup B2, were placed within the B2/ST131 portion of the tree and were clearly separated from non-ST131 lineages. Other STs formed distinct branches across the tree, reflecting the heterogeneous genetic backgrounds of MDR *E. coli* carried by healthy dogs in this collection.

To further explore the evolutionary context of the ST131 lineage identified in this study, the two canine ST131 isolates were compared with publicly available genomes belonging to the same ST retrieved from EnteroBase. After quality filtering and removal of genomes lacking epidemiological metadata, a total of 170 publicly available ST131 genomes from South American countries and diverse host sources were included in the comparative dataset. The two canine ST131 isolates characterized in this study and two reference genomes, EC958 and NCTC13441, were additionally incorporated for phylogenetic inference. Metadata and EnteroBase assembly identifiers of the 170 publicly available ST131 genomes included in the comparative analysis are provided in Supplementary Table S2.

**Figure 5:**
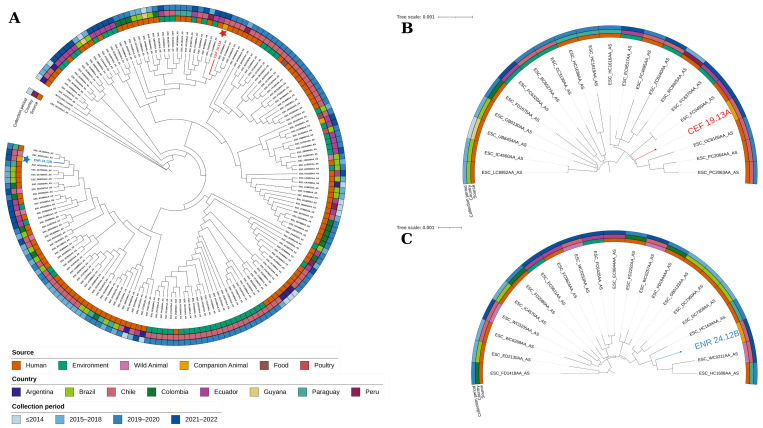
Maximum-likelihood phylogeny inferred from a cgSNP alignment of 174 *E. coli* ST131 genomes, comprising 170 publicly available genomes from South America, two canine isolates characterized in this study, and two reference genomes (EC958 and NCTC13441). Outer rings in panel (**A**) indicate source, country of origin, and collection period, with colors corresponding to the metadata categories shown in the figure. Asterisks in panel (**A**) indicate the positions of the two Chilean canine ST131 isolates generated in this study. Isolate CEF 19.13A is highlighted in red and isolate ENR 24.12B in blue; the same colors are retained in panels (**B**) and (**C**), respectively. Panels (**B**) and (**C**) show enlarged views of the clades containing CEF 19.13A and ENR 24.12B, illustrating their relative phylogenetic positions among the closest genomes included in the dataset.

Within the South American comparative ST131 phylogeny, the two canine isolates clustered within distinct ST131 subclades (Figure 5). Isolate CEF 19.13A was assigned to the C2/H30Rx lineage, whereas ENR 24.12B clustered within the C1-M27 lineage. In this comparative phylogeny, the two Chilean canine ST131 isolates were placed within a broader South American host-mixed ST131 population that included human, poultry, environmental, and other sources, rather than within an exclusive dog-associated cluster.

To assess the relatedness of the Chilean canine ST131 isolates within the comparative dataset, pairwise SNP distances were calculated from the core genome alignment. The complete pairwise SNP distance matrix is provided in Supplementary Data S1. Although both isolates belonged to ST131, CEF 19.13A and ENR 24.12B differed by 273 core-genome SNPs from each other. By contrast, each isolate showed closer relationships to distinct publicly available genomes in the comparative dataset, with distances of 38–71 SNPs for CEF 19.13A and 78–103 SNPs for ENR 24.12B to their respective ten nearest neighbors (Supplementary Table S3). These distances were interpreted as measures of relative genomic relatedness within the comparative ST131 dataset, rather than as evidence of direct transmission. The 273 cgSNP distance between the two Chilean canine ST131 isolates indicates that they do not represent a recent shared local clonal cluster, whereas the lower distances to distinct public genomes indicate closer phylogenetic affinity to different South American ST131 backgrounds. The ten closest genomes to CEF 19.13A originated from Ecuador, Peru, Chile, Paraguay, Guyana and Brazil and were predominantly human (8/10), with the remainder from environmental sources (2/10). In turn, the ten closest genomes to ENR 24.12B originated from Paraguay, Brazil, Chile, Ecuador and Colombia and included mainly human genomes (7/10), together with environmental (2/10) and wild-animal (1/10) genomes. Together, the phylogenetic reconstruction and pairwise SNP analysis indicate that the two canine isolates occupy distinct regions of the ST131 phylogeny rather than forming a single shared local canine cluster.

## 4. Discussion

This study provides genomic and phenotypic characterization of 13 genetically non-redundant MDR *E. coli* isolates selected from a previously described surveillance collection obtained from clinically healthy dogs in urban veterinary settings in Chile. Although the study was not designed to estimate prevalence, it offers a targeted genomic description of diverse resistant lineages detected in this host population, including ESBL-associated backgrounds, diverse plasmid replicons, and ST131 subclades of recognized epidemiological interest. Collectively, these findings expand the still limited genomic evidence on MDR *E. coli* carried by healthy dogs in Latin America and support further consideration of companion animals in genomic AMR surveillance efforts.

The detection of ESBL-producing isolates and the identification of ST131 among healthy dogs reinforce growing evidence that companion animals can participate in the ecology of antimicrobial-resistant *E. coli* in urban environments [18,22,24]. These findings add genomic evidence on ST131 among *E. coli* isolated from clinically healthy dogs in Chile and contribute to the still limited regional dataset on companion-animal AMR. Importantly, none of the dogs included in this study had a recent history of antimicrobial treatment, as reported in the original surveillance study [29], suggesting that resistant *E. coli* may still be detected in the canine intestinal microbiota in the absence of recent direct antimicrobial exposure.

The resistance patterns observed here are consistent with international reports showing that healthy dogs may carry MDR *E. coli* even in the absence of clinical disease. Previous Chilean studies provide an important context for interpreting these findings. In small-scale farms of central Chile, ESBL-producing *E. coli* carrying *bla*_CTX-M_ genes were detected among livestock, dogs and wild mammals, suggesting circulation of ESBL-producing *E. coli* across domestic, livestock and wildlife interfaces [28]. In urban household dogs from the Metropolitan Region, previous studies based on the same surveillance framework documented phenotypic and genotypic antimicrobial resistance in *E. coli*, including the parent collection from which the isolates analyzed here were selected, and identified epidemiological risk factors associated with carriage of critical antimicrobial-resistant *E. coli* [13,29]. The present study builds directly on this Chilean urban-dog surveillance framework by adding WGS-based characterization of a selected non-redundant subset of resistant canine *E. coli*, allowing assessment of STs, plasmid replicons, virulence-associated genes and ST131 subclades. High resistance to aminopenicillins and first- and third-generation cephalosporins is in line with the widespread dissemination of ESBL and plasmid-mediated AmpC determinants in companion animals [27,50]. Fluoroquinolone resistance was also prominent, which is especially relevant given the critical importance of this antimicrobial class in human medicine and its frequent use in small animal clinical practice [51,52,53]. Previous studies from Europe have reported resistance to third-generation cephalosporins in canine *E. coli* in the order of 30–60%, whereas fluoroquinolone resistance has generally ranged from 40% to 70%, depending on study design and sampling strategy [27]. Reports from Latin America, including Chile, Brazil, Argentina and Ecuador, have documented ESBL/AmpC-producing and multidrug-resistant *E. coli* in companion animals, including fecal isolates from dogs, indicating that these phenotypes are not restricted to overt infection and may also be established in commensal populations [29,54,55,56,57]. In this context, the MAR indices observed here are consistent with broad resistance across multiple antimicrobial classes among the selected isolates.

At the genomic level, the predominance of *bla*_CTX-M_ variants and the frequent detection of IncF-associated plasmid replicons mirror findings reported in both human and animal *E. coli* populations worldwide [58,59]. The predominance of IncF-associated plasmid replicons, together with the co-occurrence of determinants associated with β-lactam, fluoroquinolone, tetracycline and sulfonamide resistance, underscores the central role of mobile genetic elements in shaping the canine resistome. This is epidemiologically relevant because these plasmid platforms are well recognized for facilitating the persistence and dissemination of MDR across bacterial lineages and host species [19,58,60]. In particular, IncF plasmids have been widely implicated in the evolutionary success of ST131 clade C through successful clone–plasmid combinations carrying *bla*_CTX-M_ determinants and other resistance traits [19,61].

The detection of ST131 in this selected canine panel deserves particular attention because this lineage is globally distributed across human populations and has increasingly been reported in companion animals, including both clinical and fecal carriage contexts. From an evolutionary perspective, ST131 is especially relevant because it was initially recognized as a human-associated ExPEC lineage and subsequently emerged as a globally successful pandemic clone through the stepwise expansion of specific subclades linked to fluoroquinolone resistance and ESBL acquisition [18,62,63]. Large-scale genomic analyses of a worldwide ST131 collection showed that the global success of this lineage was largely driven by the expansion of clade C/H30 descendants, particularly lineages defined by characteristic QRDR mutations and later by acquisition of epidemic ESBL platforms such as *bla*_CTX-M-15_ [63]. Within this framework, the C2/H30Rx subclade became strongly associated with *bla*_CTX-M-15_ and widespread dissemination in human extraintestinal infections, whereas the more recently emerged C1-M27 lineage was first recognized as a major driver of ESBL-producing ExPEC in Japan and had already intercontinentally spread by the time of its initial global description [64,65,66].

Within this ST131 framework, the identification in clinically healthy dogs of one isolate assigned to C2/H30Rx and another to C1-M27 is notable because both subclades have previously been reported in companion animals. This finding is epidemiologically relevant because dogs live in close and prolonged contact with their human caregivers. Companion animals have also been shown to act as spillover hosts or household reservoirs of ST131 and other extraintestinal pathogenic *E. coli* lineages, although direct transmission cannot be inferred from the present study [22,26]. In Australia, a nationwide study of fluoroquinolone-resistant ExPEC from dogs and cats identified 20 ST131 isolates, of which 65% belonged to clade C/H30, including C1/H30R1 and C2/H30Rx subclones [22]. In Japan, whole-genome analysis of ESBL/AmpC-producing *E. coli* from companion dogs identified six ST131 isolates among 19 canine isolates, including four O25:H4/*fimH*30 C1-M27 strains carrying *bla*_CTX-M-27_ on IncFIA/FIB/FII plasmids and showing close chromosomal similarity to human ST131/C1-M27 strains [24]. In addition, the emergence of the C1-M27 cluster has also been documented in companion animals in France [67]. Comparative genomic studies further indicate that canine ST131 isolates often intermingle with human-associated diversity, although some human–canine associated subclusters have also been described [22,68]. In that context, our data are more consistent with the placement of the Chilean canine isolates within a broader South American host-mixed ST131 population than with a clearly segregated canine-only ST131 cluster. This interpretation is supported by the phylogenomic placement of the Chilean isolates among genomes from human, poultry and environmental sources and by the fact that the two Chilean ST131 isolates were more closely related to different publicly available genomes than to each other. Consistently, nearest-neighbor SNP comparisons showed that both Chilean ST131 isolates were more closely related to distinct publicly available genomes from multiple South American countries and from predominantly human, but also environmental and wild-animal sources, rather than to a shared local canine cluster. Although none of the closest genomes to either isolate corresponded to companion-animal sources, this finding should be interpreted cautiously given the composition of the comparative dataset. Accordingly, these results are consistent with the view that dogs may participate in the maintenance and circulation of globally distributed ST131 lineages in community settings, although the directionality of transmission cannot be inferred from this study.

The pairwise SNP distances are informative in this regard, but they should be interpreted cautiously. No universal SNP threshold can be applied across all *E. coli* ST131 datasets to define clonality or transmission because interpretation depends on sampling density, temporal scale, epidemiological context, recombination filtering, and the analytical pipeline used. In this study, SNP distances were therefore used to assess relative genomic relatedness within the comparative ST131 dataset rather than to infer direct transmission. The two Chilean canine ST131 isolates differed by 273 cgSNPs, supporting the view that they do not represent a recent shared local clonal cluster. By contrast, the lower distances observed between each canine isolate and distinct publicly available genomes indicate closer phylogenetic affinity to different South American ST131 backgrounds. However, these distances remain insufficient to infer direct epidemiological linkage in the absence of household, temporal, source-tracing, or contact data.

Other sequence types identified here, including ST1011, ST744 and ST1196, further highlight the genetic heterogeneity of MDR *E. coli* circulating in dogs [69,70]. However, unlike ST131, their detection should not be taken as evidence that these isolates represent confirmed ExPEC lineages in this isolate set. For fecal isolates, ExPEC assignment is usually based on operational molecular criteria rather than on isolated virulence markers alone. Under the widely used Johnson definition, an isolate is classified as ExPEC when it carries at least two of five hallmark markers, namely *pap*, *sfa*/*foc*, *afa*/*dra*, *kpsMII* and *iutA*; for urovirulence-oriented classification, the Spurbeck scheme emphasizes *fyuA* together with at least two of *chuA*, *yfcV* and *vat* [71,72]. Applying these criteria to the present dataset, 5/13 isolates (38.5%) fulfilled the Johnson molecular ExPEC criterion, whereas 3/13 isolates (23.1%) fulfilled the Spurbeck urovirulence-oriented criterion. The three isolates meeting the Spurbeck criterion were also included among those fulfilling the Johnson criterion. These classifications should be interpreted as molecular evidence of ExPEC- or urovirulence-associated potential, rather than as functional confirmation of extraintestinal pathogenicity. In the present study, several non-ST131 isolates carried virulence-associated genes commonly reported in ExPEC, particularly adhesins, iron acquisition systems and selected serum survival-associated loci, but this pattern alone is insufficient to confirm them as ExPEC. A more appropriate interpretation is that these isolates harbor ExPEC-associated traits and broader fitness- or accessory virulence-associated determinants of potential epidemiological relevance. This distinction is important because many genes frequently discussed in relation to ExPEC, particularly adhesins, iron acquisition systems and other fitness-associated loci, may also occur in commensal intestinal *E. coli*. Moreover, fecal carriage itself may represent the reservoir from which extraintestinal lineages emerge [16,73]. Even so, the coexistence of virulence-associated determinants with MDR remains epidemiologically relevant in healthy dogs, especially for B2 isolates and for lineages carrying broader accessory virulence repertoires [69,71,72,73].

From a One Health perspective, these findings underscore the still underrecognized role of companion animals in the ecology of AMR. Despite close human–animal interactions, dogs remain underrepresented in most surveillance systems, which have historically prioritized humans and food-producing animals [3,8,27]. This gap is particularly evident in Chile and Latin America, where genomic data on AMR in companion animals remain limited [13,29]. The detection in healthy dogs of globally distributed resistance determinants, IncF-associated plasmid backgrounds and pandemic ST131 subclades is consistent with the potential involvement of companion animals in broader AMR ecology across human, animal and environmental interfaces. Incorporating companion animals into surveillance frameworks could therefore help strengthen regional capacity to detect, interpret and mitigate the circulation of clinically relevant resistant bacteria within a One Health approach [8,27].

Despite the novelty of our results, this study has some limitations. First, the number of isolates subjected to WGS was limited, and the selection strategy was designed to reduce redundancy and explore genetic diversity rather than to estimate the frequency of MDR lineages in the broader canine population. Accordingly, these findings should not be interpreted as prevalence estimates for healthy dogs in Chile or as a proportional representation of the 224 resistant *E. coli* isolates from the parent collection. The proportion of total genomic diversity captured by this subset therefore cannot be formally quantified from the present design. Second, the sequenced isolates were selected from a resistant collection originally recovered on antibiotic-supplemented media, which enriched for resistant phenotypes and further limits extrapolation of resistance frequencies to the wider canine fecal *E. coli* population. Third, although the phylogenomic analysis included a diverse comparative ST131 dataset, broader comparisons with larger international collections may further refine the evolutionary placement and epidemiological context of the canine isolates. Nevertheless, these limitations do not diminish the relevance of the present findings. Rather, they position this study as targeted genomic evidence that clinically healthy dogs in Chile can harbor MDR *E. coli* carrying clinically relevant resistance determinants, virulence-associated traits, and ST131 subclades of recognized epidemiological interest. Taken together, the results support the inclusion of companion animals in integrated One Health surveillance strategies.

## 5. Conclusions

This study provides a diversity-oriented genomic snapshot of selected MDR *E. coli* isolates recovered from clinically healthy dogs in the Metropolitan Region of Chile, including two ST131 isolates assigned to the C2/H30Rx and C1-M27 subclades. The coexistence of *bla*_CTX-M_ variants, IncF-associated plasmid replicons, and virulence-associated traits in selected isolates suggests that clinically healthy dogs may carry resistant *E. coli* lineages of potential epidemiological relevance. Although this study does not estimate prevalence or demonstrate direct transmission, the phylogenomic placement of the canine ST131 isolates within a comparative South American dataset is compatible with, but does not directly demonstrate, shared AMR ecology across human, animal, and environmental sectors. These findings support further consideration of companion animals in integrated genomic AMR surveillance programs in Chile and Latin America.

## Figures and Tables

**Table 1:** Metadata, genomic classification and resistance phenotypes of the 13 selected *E. coli* isolates recovered from clinically healthy dogs in the Metropolitan Region of Chile.

Isolate ID	Geographic Sector	Year of Isolation	ST	Phylogroup	Serotype	*fimH*	Resistance Phenotype
AMX 16A	Northeast	2021	491	B2	O131:H45	*fimH5*	AMP, CLE, CPO, CIP, ENR, MAR, DOX, CHL, TRS
AMX 22.27A	North	2022	1196	B1	O91:H28	*fimH31*	AMP, AMC, CLE, GEN, CIP, ENR, MAR, DOX
AMX 27.5A	Southwest	2022	1011	E	O-:H16	*fimH31*	AMP, CLE, CPO, CVN, CTF, CIP, ENR, MAR, TRS
CEF 16B	Northeast	2021	162	B1	O8:H19	*fimH32*	AMP, CLE, CPO, CVN, CTF, CIP, ENR, MAR, CHL, TRS
CEF 3.14A	Northeast	2021	224	B1	O-:H23	*fimH39*	AMP, CLE, CPO, CVN, CTF, CIP, ENR, MAR, DOX
CEF 5.5A	Center	2022	744	A	O101:H9	*fimH54*	AMP, CLE, CPO, CVN, CTF, CIP, ENR, MAR, DOX, CHL
CEF 19.13A	Center	2022	131	B2	O25:H4	*fimH30*	AMP, AMC, CLE, CPO, CVN, CTZ, CTF, GEN, CIP, ENR, MAR, TRS
CEF 20.4A	North	2022	10	A	O101:H9	*fimH54*	AMP, AMC, CLE, CPO, CVN, CTZ, CTF, CIP, TRS
ENR 8.7A	Southeast	2021	90	C	O8:H9	*fimH142*	AMP, AMC, CLE, GEN, CIP, ENR, MAR, TRS
ENR 16.16B	Southeast	2022	1196	B1	O-:H28	*fimH31*	AMP, AMC, CLE, CPO, CVN, CTF, CIP, ENR, MAR
ENR 16.18B	Southeast	2022	1011	D	O85:H16	*fimH31*	AMP, CLE, CPO, CVN, CTF, CIP, ENR, MAR, TRS
ENR 18.6A	South	2022	224	B1	O29:H23	*fimH61*	AMP, AMC, CLE, CPO, CVN, CTF, GEN, CIP, ENR, MAR, DOX, CHL, TRS
ENR 24.12B	Center	2022	131	B2	O25:H4	*fimH30*	AMP, AMC, CLE, CPO, CVN, CTF, GEN, CIP, ENR, MAR, DOX

Geographic sector corresponds to the macro-area of the Metropolitan Region where the veterinary clinic was located. ST, sequence type according to the Achtman MLST scheme. Phylogroup assignment was performed using the Clermont typing method. *fimH* allele designation was assigned using FimTyper. Resistance phenotype includes only antimicrobials classified as resistant according to phenotypic antimicrobial susceptibility testing. Antimicrobial abbreviations: AMP, ampicillin; AMC, amoxicillin/clavulanic acid; CLE, cephalexin; CPO, cefpodoxime; CVN, cefovecin; CTZ, ceftazidime; CTF, ceftiofur; GEN, gentamicin; CIP, ciprofloxacin; ENR, enrofloxacin; MAR, marbofloxacin; DOX, doxycycline; CHL, chloramphenicol; TRS, trimethoprim/sulfamethoxazole.

## Data Availability

The genomic data generated in this study have been deposited in NCBI under BioProject PRJNA1447459. BioSample accessions SAMN56978300-SAMN56978312 have been assigned for the 13 isolates included in this study. All other relevant data are contained within the article, its Supplementary Materials, and Supplementary Data S1.

## Supplementary Materials

The following supporting information can be downloaded at: https://www.mdpi.com/article/10.3390/ani16121769/s1, Supplementary Table S1. Available dog-level metadata, assembly statistics, phenotypic antimicrobial susceptibility results, and curated genomic features of the 13 canine *Escherichia coli* isolates included in this study; Supplementary Table S2. Metadata and EnteroBase assembly identifiers of the publicly available ST131 genomes included in the comparative phylogenomic analysis; Supplementary Table S3. Pairwise core-genome SNP distances between the Chilean canine ST131 isolates and their ten nearest neighbors in the comparative dataset; Supplementary Table S4. Genotype–phenotype concordance between antimicrobial resistance profiles and detected resistance determinants in the 13 canine *Escherichia coli* isolates; Supplementary Data S1. Raw pairwise SNP distance matrix generated from the ST131 comparative core-genome alignment.

## Abbreviations

The following abbreviations are used in this manuscript:

AMR	Antimicrobial resistance
ARGs	Antimicrobial resistance genes
cgSNP	Core-genome single-nucleotide polymorphism
CLSI	Clinical and Laboratory Standards Institute
ECDC	European Centre for Disease Prevention and Control
EFSA	European Food Safety Authority
ESBL	Extended-spectrum β-lactamase
ExPEC	Extraintestinal pathogenic *Escherichia coli*
MAR	Multiple antimicrobial resistance
MDR	Multidrug-resistant
MIC	Minimum inhibitory concentration
MLST	Multilocus sequence typing
PMQR	Plasmid-mediated quinolone resistance
QRDR	Quinolone resistance-determining region
SNP	Single-nucleotide polymorphism
ST	Sequence type
WGS	Whole-genome sequencing
WHO	World Health Organization
